# Overall asthma control achieved with budesonide/formoterol maintenance and reliever therapy for patients on different treatment steps

**DOI:** 10.1186/1465-9921-12-38

**Published:** 2011-04-04

**Authors:** Eric D Bateman, Tim W Harrison, Santiago Quirce, Helen K Reddel, Roland Buhl, Marc Humbert, Christine R Jenkins, Stefan Peterson, Ollie Östlund, Paul M O'Byrne, Malcolm R Sears, Göran S Eriksson

**Affiliations:** 1 Division of Pulmonology, Department of Medicine, University of Cape Town, Cape Town, South Africa; 2 Nottingham Biomedical Research Unit, City Hospital Campus, Nottingham University, Nottingham, UK; 3 Department of Allergy, Hospital La Paz, Universidad Autónoma de Madrid, Madrid, Spain; 4 Clinical Management Group, Woolcock Institute of Medical Research, Sydney, Australia; 5 Pulmonary Department, Mainz University Hospital, Mainz, Germany; 6 Université Paris-Sud 11, Centre National de Référence de L'Hypertension Artérielle Pulmonaire, Service de Pneumologie et Réanimation Respiratoire, Hôpital Antoine-Béclère, Clamar Cedex, France; 7 AstraZeneca Research and Development, Lund, Sweden; 8 Michael G DeGroote School of Medicine, Faculty of Health Sciences, McMaster University, Hamilton, Ontario, Canada; 9 Department of Respiratory Medicine and Allergology, University Hospital, Lund, Sweden

## Abstract

**Background:**

Adjusting medication for uncontrolled asthma involves selecting one of several options from the same or a higher treatment step outlined in asthma guidelines. We examined the relative benefit of introducing budesonide/formoterol (BUD/FORM) maintenance and reliever therapy (Symbicort SMART^® ^Turbuhaler^®^) in patients previously prescribed treatments from Global Initiative for Asthma (GINA) Steps 2, 3 or 4.

**Methods:**

This is a *post hoc *analysis of the results of five large clinical trials (>12000 patients) comparing BUD/FORM maintenance and reliever therapy with other treatments categorised by treatment step at study entry. Both current clinical asthma control during the last week of treatment and exacerbations during the study were examined.

**Results:**

At each GINA treatment step, the proportion of patients achieving target levels of current clinical control were similar or higher with BUD/FORM maintenance and reliever therapy compared with the same or a higher fixed maintenance dose of inhaled corticosteroid/long-acting β_2_-agonist (ICS/LABA) (plus short-acting β_2_-agonist [SABA] as reliever), and rates of exacerbations were lower at all treatment steps in BUD/FORM maintenance and reliever therapy versus same maintenance dose ICS/LABA (P < 0.01) and at treatment Step 4 versus higher maintenance dose ICS/LABA (P < 0.001). BUD/FORM maintenance and reliever therapy also achieved significantly higher rates of current clinical control and significantly lower exacerbation rates at most treatment steps compared with a higher maintenance dose ICS + SABA (Steps 2-4 for control and Steps 3 and 4 for exacerbations). With all treatments, the proportion of patients achieving current clinical control was lower with increasing treatment steps.

**Conclusions:**

BUD/FORM maintenance and reliever therapy may be a preferable option for patients on Steps 2 to 4 of asthma guidelines requiring a more effective treatment and, compared with other fixed dose alternatives, is most effective in the higher treatment steps.

## Background

A major objective of the 2006 revision of the Global Initiative for Asthma (GINA) guidelines [1] was to simplify the process of assessing patients' treatment needs at both initial and follow-up visits. Instead of assessing "asthma severity" using severity classification tables, a simplified assessment of current asthma control is recommended and treatment is either initiated or altered according to the assessed control status. This approach has, with some modification, been adopted in most international and recent versions of national treatment guidelines and has been well received by practising clinicians [2]. Central to this approach are the treatment steps defining initial maintenance treatment and recommendations for stepping up or stepping down treatment. These are based on treatment response - whether or not current clinical control is achieved and maintained. Thus, patients failing Step 2 treatment, on a low dose of inhaled corticosteroid (ICS), might be moved up to Step 3 where the preferred option for adolescents and adults is the addition of a long-acting β_2_-agonist (LABA). At Step 4, an increase in ICS dose or the addition of another controller medication (e.g. a leukotriene modifier or sustained-release theophylline) is recommended in order to achieve control. However, in the significant proportion of patients in which control is not achieved, Step 5 management may be considered, recognising that lack of control is associated with a higher risk of exacerbations, poor quality of life and other adverse outcomes [2].

For patients whose asthma is uncontrolled on Step 2 or higher treatment, a recent strategy included in GINA guidelines is the use of a combination inhaler containing budesonide and formoterol for both maintenance therapy and relief of symptoms (option for Steps 3-5). This approach has been shown in several large-scale randomised controlled trials to achieve similar current clinical control of asthma and to be superior to comparators in reducing asthma exacerbations [3-7]. These results have been achieved with the use of significantly lower doses of inhaled and systemic corticosteroids [3-6]. The comparators in these studies have been either a two- to four-fold higher dose of budesonide (a Step 2-3 option) [3,4], the same dose of ICS plus a LABA (a Step 3 option) or a higher-dose ICS plus a LABA (a Step 4 treatment), together with a short-acting β_2_-agonist (SABA) for as-needed relief of symptoms [3,5-7]. Recent *post hoc *analyses of the results of these studies have provided further insights into the relationship between levels of current asthma control achieved and "future risk" [8-10]. From the clinician's perspective, the decision to consider a change in medication will usually be prompted by a patient's failure to achieve control on current treatment (Steps 2-4). It is therefore appropriate to examine the relative benefit of introducing budesonide/formoterol (BUD/FORM) maintenance and reliever therapy in patients previously prescribed different GINA steps of treatment. We report here a *post hoc *analysis of the results of five clinical trials comparing BUD/FORM maintenance and reliever therapy with comparator treatments when introduced in patients on different treatment steps (Steps 2-4) according to GINA guidelines at study entry.

## Methods

### Objectives

This pooled analysis describes the relative effect of BUD/FORM maintenance and reliever therapy on current asthma control and on exacerbation risk, compared with fixed-dose maintenance regimens of ICS or combination ICS/LABA, plus SABA as reliever, in patients with asthma stratified by GINA treatment step at study entry.

### Studies and population

The clinical studies included in this retrospective analysis involved >12000 patients from five double-blind, randomised, parallel-group trials (6-12 months' duration) with exacerbation as a primary variable. The trials investigated the efficacy of BUD/FORM maintenance and reliever therapy (Symbicort SMART^® ^Turbuhaler^®^, AstraZeneca AB, Lund, Sweden) versus comparator therapies which included: higher maintenance dose ICS (budesonide) plus SABA as needed [3,4]; same maintenance dose ICS/LABA (BUD/FORM, Symbicort^®^, AstraZeneca, Lund, Sweden) plus SABA as needed [3,7]; and higher maintenance dose ICS/LABA (BUD/FORM [5] or salmeterol/fluticasone [Seretide™, GlaxoSmithKline, Uxbridge, UK]) plus SABA as needed [5,6]. In all studies the SABA used was terbutaline (Bricanyl^®^, AstraZeneca, Sweden; 0.4 mg/inhalation) and all aforementioned drugs were administered via Turbuhaler^® ^(AstraZeneca, Lund, Sweden) except for salmeterol/fluticasone which was delivered via either Diskus™ or Evohaler™ (GlaxoSmithKline, Uxbridge, UK).

The methodologies of the five studies have been published in detail previously [3-7]. Further details of each of these studies are provided in the additional files (see Additional file 1, Table S1 of the additional files). All five studies were performed in accordance with the Declaration of Helsinki and Good Clinical Practice guidelines and were approved by independent ethics committees. Written informed consent was obtained from each adult patient; for patients under 18 years of age, informed consent was obtained from both the patient and his/her legal guardian. As this retrospective analysis is based on these five studies no additional approvals were applied for.

#### Stratification by GINA treatment step at entry

Patients with asthma at least 12 years of age who were symptomatic during run-in were randomised. Data collected at study entry were used to classify each patient's pre-study asthma medication as Step 2, 3 or 4 according to GINA classification based on ICS dose and use of other controller medication. In all studies the regular use of ICS was an inclusion criterion but use of systemic steroids within 30 days of study entry was an exclusion criterion - thus patients on either Step 1 or Step 5 were excluded[3-7]. The GINA treatment steps were defined as:

• Step 2 - low-dose ICS

• Step 3 - low-dose ICS plus at least one of a LABA, a leukotriene receptor antagonist (LTRA) or xanthine, or medium- to high-dose ICS alone

• Step 4 - medium- to high-dose ICS plus at least one of a LABA (including high-dose ICS/LABA combination), an LTRA or xanthine (Additional file 1, Tables S2 and S3).

### Assessments

#### Asthma control as defined by GINA criteria

Each patient's asthma control, defined by GINA guideline-derived criteria (Controlled, Partly Controlled or Uncontrolled asthma) [2], was determined retrospectively each week from diary data in the studies. In this report, we have considered only the results from the last week of the run-in period and the last week of the study [9]. Where there was insufficient diary data to perform an assessment of control, the week was considered Uncontrolled.

#### FEV_1_

Forced expiratory volume in 1 second (FEV_1_) was recorded at clinic visits and the percentage of predicted normal was calculated. At the study entry visit pre- and post-bronchodilator FEV_1 _was recorded and for all subsequent visits patients were requested to take their morning study medication as usual.

#### Reliever use

Reliever use per patient per 24 hours was derived from diary cards and summarised as mean over the last 10 days of run-in and over the study period.

#### Exacerbations

Due to minor differences in the definition of exacerbations across the original studies, the definition of exacerbations in this pooled analysis was standardised to a worsening of asthma symptoms requiring an oral steroid course of at least 3 days' duration, an emergency room (ER) visit or a hospitalisation for treatment of asthma.

For more details of the definitions of control and exacerbations please refer to the additional files.

### Statistical methods

The main purpose of the analysis was to estimate the effect of BUD/FORM maintenance and reliever therapy versus a comparator for each individual GINA treatment step at entry. P-values below 5% were regarded as indicating statistical significance.

The percentage of patients in each control state during the last study week was presented by treatment and GINA treatment step and analysed in two separate logistic regression models - one for the odds of being Controlled and one for the odds of being at least Partly Controlled - both with factors including treatment, GINA treatment step, treatment-GINA treatment step interaction and study. The number of exacerbations was presented as yearly exacerbation rates by GINA treatment step and treatment and analysed using a Poisson model with factors including treatment, GINA treatment step, treatment-GINA treatment step interaction and study, and observation time as an offset, with adjustments performed for overdispersion if present. Time to first exacerbation was described by Kaplan-Meier plots stratified by treatment and GINA treatment step and analysed in a Cox proportional hazard model with factors including treatment, GINA treatment step and treatment-GINA treatment step at entry interaction, stratified by study. The same models were applied to hospitalisations and ER treatments.

The change in FEV_1 _as percentage of predicted normal, from randomisation to last visit on treatment, was analysed using analysis of variance (ANOVA) with the same factors as above and with the value at randomisation as a covariate. In calculating mean FEV_1 _over time by GINA treatment step and treatment group the last observation carried forward principle was used to correct for missing values. In the plot of this data, as visits occurred at different intervals in the studies, values were positioned with the month nearest to the study visit. Thus the 1- and 3-month-visit data from the Bousquet *et al. *study were plotted with the 2- and 4-month-visit data from the Kuna *et al. *study, and the 4- and 8-month-visit data from the Rabe *et al. *study were plotted with the 3- and 9-month data from the O'Byrne *et al. *and the Scicchitano *et al. *study, respectively [3-7]. In addition, a 6-month value was imputed for patients in the Rabe *et al. *study as the mean of the 4- and 8-month-visit values [7].

Patient means of daily reliever use during the treatment period were analysed using ANOVA analysis of variance with the same factors as above and with mean reliever use during run-in as a covariate.

## Results

### GINA treatment steps at entry and demographics

A total of 12512 subjects were included in the analysis described; only one patient included in the original trials lacked data on pre-entry medication and was excluded from the analysis. The numbers of patients defined as being at GINA treatment Steps 2, 3 or 4 at entry were 1037, 6352 and 5123 respectively. All patients used ICS, 45% used LABA, 5% used theophylline, 3% used LTRAs and ≤ 2% used anticholinergics, cromones or oral β_2_-agonists (see Additional file 1, Table S3 in additional files).

Baseline characteristics were similar between comparator treatment groups in this pooled analysis (Table 1). By categorising patients by their GINA treatment step at entry, some differences were evident between the steps for patient age and FEV_1 _but mean total symptom scores were similar.

**Table 1:** Baseline characteristics of study subjects

Budesonide/formoterol maintenance and reliever therapy vs. higher maintenance dose ICS + SABA						
	**Step 2**		**Step 3**		**Step 4**	

	Higher maintenance ICS + SABA (N = 262)	BUD/FORM maintenance and reliever therapy (N = 231)	Higher maintenance ICS + SABA (N = 1013)	BUD/FORM maintenance and reliever therapy (N = 1051)	Higher maintenance ICS + SABA (N = 769)	BUD/FORM maintenance and reliever therapy (N = 747)

Male, n (%)	123 (47)	104 (45)	399 (39)	433 (41)	318 (41)	315 (42)

Age, mean	30.6	32.2	39.5	37.5	42.6	42.0

ICS, n (%) Low	262 (100)	231 (100)	242 (24)	226 (22)	0	0

Medium	0	0	664 (66)	711 (68)	643 (84)	591 (79)

High	0	0	107 (11)	114 (11)	126 (16)	156 (21)

LABA use, n (%)	0	0	221 (22)	214 (20)	706 (92)	689 (92)

Asthma diagnosis, median years	6.5	9	10	9	10	9

FEV_1_, % PN	74.3	74.5	72.5	72.8	71.2	72.3

As-needed use, mean daily	2.16	2.24	2.03	2.08	2.26	2.12

Symptom score, mean total	1.36	1.37	1.57	1.63	1.74	1.65

**Budesonide/formoterol maintenance and reliever therapy vs. same maintenance dose ICS/LABA + SABA**						

	**Step 2**		**Step 3**		**Step 4**	

	Same maintenance ICS/LABA + SABA (N = 375)	BUD/FORM maintenance and reliever therapy (N = 389)	Same maintenance ICS/LABA + SABA (N = 896)	BUD/FORM maintenance and reliever therapy (N = 883)	Same maintenance ICS/LABA + SABA (N = 596)	BUD/FORM maintenance and reliever therapy (N = 597)

Male, n (%)	157 (42)	170 (44)	389 (43)	391 (44)	274 (46)	250 (42)

Age, mean	35.3	34.7	39.4	37.7	42.6	43.4

ICS, n (%) Low	375 (100)	389 (100)	130 (15)	136 (15)	0	0

Medium	0	0	671 (75)	644 (73)	415 (70)	411 (69)

High	0	0	95 (11)	103 (12)	181 (30)	186 (31)

LABA use, n (%)	0	0	107 (12)	118 (13)	548 (92)	548 (92)

Asthma diagnosis, median years	8	9	11	10	12	13

FEV_1_, % PN	72.5	73.7	71.9	72.1	70.8	70.3

As-needed use, mean daily	2.01	1.98	2.17	2.18	2.34	2.23

Symptom score, mean total	1.62	1.56	1.68	1.65	1.78	1.76

**Budesonide/formoterol maintenance and reliever therapy vs. higher maintenance dose ICS/LABA + SABA**						

			Step 3*		**Step 4**	
			
			Higher maintenance ICS/LABA + SABA (N = 1786)	BUD/FORM maintenance and reliever therapy (N = 1203)	Higher maintenance ICS/LABA + SABA (N = 1585)	BUD/FORM maintenance and reliever therapy (N = 1051)
			
Male, n (%)			716 (40)	498 (41)	654 (41)	420 (40)
			
Age, mean			36.2	36.3	40.9	41.7
			
ICS, n (%) Low			124 (7)	139 (12)	0	0
			
Medium			1424 (80)	918 (76)	1283 (81)	876 (83)
			
High			238 (13)	146 (12)	302 (19)	175 (17)
			
LABA use, n (%)			119 (7)	138 (11)	1527 (96)	1004 (96)
			
Asthma diagnosis, median years			11	11	10	12
			
FEV_1_, % PN			72.7	71.9	71.4	70.4
			
As-needed use, mean daily			2.28	2.26	2.34	2.27
			
Symptom score, mean total			1.95	1.91	1.88	1.88

BUD/FORM = budesonide/formoterol; FEV_1 _= forced expiratory volume in 1 second; ICS = inhaled corticosteroid; LABA = long-acting β_2_-agonist; PN = predicted normal; SABA = short-acting β_2_-agonist

*Seven Step 2 classified patients were pooled with Step 3

GINA steps were assigned according to treatment at study entry

### Asthma control defined by GINA criteria

**Figure 1:**
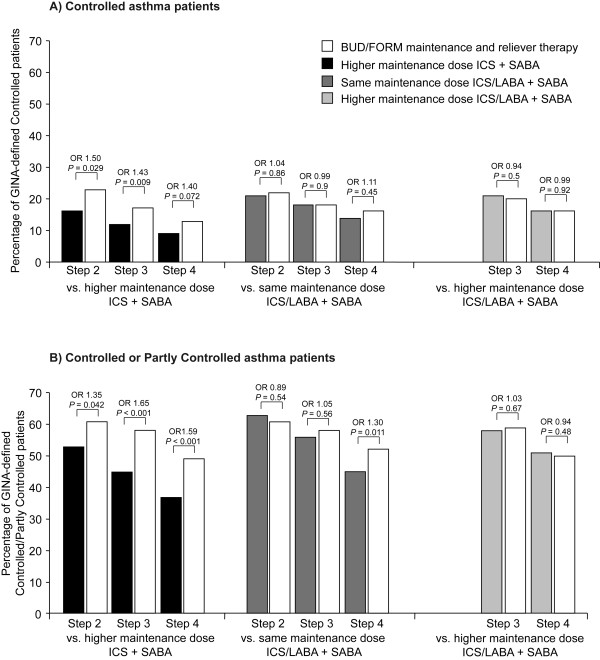
**Proportion of Controlled or Partly Controlled asthma patients in final week of treatment by study treatment and GINA treatment step at entry**. BUD/FORM = budesonide/formoterol; ICS = inhaled corticosteroid; LABA = long-acting β_2_-agonist; OR = odds ratio; SABA = short-acting β_2_-agonist

In the final week of treatment, the proportion of patients with GINA-defined Controlled asthma and Controlled or Partly Controlled asthma decreased with increasing GINA treatment step, irrespective of study treatment (Figures 1A and 1B). The proportion of patients with Controlled asthma was higher for all GINA treatment steps when BUD/FORM maintenance and reliever therapy was compared with higher maintenance dose ICS + SABA (13-23% vs. 9-16%, respectively) and was statistically significant for GINA treatment Steps 2 (P = 0.03) and 3 (P < 0.01). The proportion of patients with Controlled asthma was similar for all GINA treatment steps when BUD/FORM maintenance and reliever therapy was compared with same maintenance dose ICS/LABA + SABA (16-22% vs. 14-21%) and higher maintenance dose ICS/LABA + SABA (16-20% vs. 16-21%) (Figure 1A).

The proportion of patients with Controlled or Partly Controlled asthma was significantly higher for all GINA treatment steps when BUD/FORM maintenance and reliever therapy was compared with higher maintenance dose ICS + SABA (49-61% vs. 37-53% of patients; P < 0.05). The proportion of patients with Controlled or Partly Controlled asthma was at least similar for all treatment steps when BUD/FORM maintenance and reliever therapy was compared with same maintenance dose ICS/LABA + SABA (52-61% vs. 45-63%) and was significantly higher for GINA treatment Step 4 patients (P = 0.011). When compared with higher maintenance dose ICS/LABA + SABA the proportion of patients with Controlled or Partly Controlled asthma was similar for BUD/FORM maintenance and reliever therapy for both GINA treatment steps assessed (50-59% vs. 51-58%) (Figure 1B).

### FEV_1_

**Figure 2:**
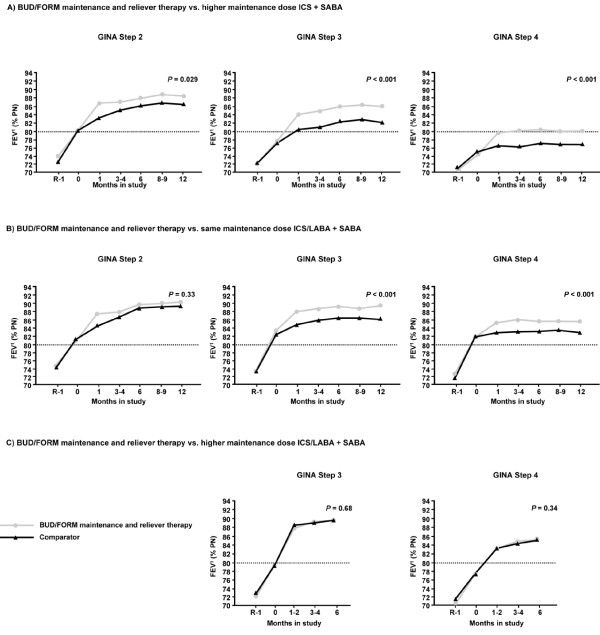
**Mean FEV**_**1 **_**by visit and GINA step at study entry.** BUD/FORM maintenance and reliever therapy vs. higher maintenance dose ICS + SABA, difference at last visit as percentage predicted normal, adjusted for FEV_1 _at randomisation visit (95% confidence interval): Step 2, 2.18 (0.22, 4.14); Step 3, 3.58 (2.29, 4.87); Step 4, 3.83 (2.25, 5.40). BUD/FORM maintenance and reliever therapy vs. same maintenance dose ICS/LABA + SABA, difference at last visit as percentage predicted normal, adjusted for FEV_1 _at randomisation visit (95% confidence interval): Step 2, 1.29 (-1.29, 3.88); Step 3, 2.50 (1.25, 3.76); Step 4, 2.70 (1.24, 4.17). BUD/FORM maintenance and reliever therapy vs. higher maintenance dose ICS/LABA + SABA, difference at last visit as percentage predicted normal, adjusted for FEV_1 _at randomisation visit (95% confidence interval): Step 3, 0.22 (-0.82, 1.26); Step 4, 0.53 (-0.57, 1.64).

On average, higher GINA treatment steps at entry were associated with a lower FEV_1 _both before and during study treatment. Following randomisation, mean FEV_1 _increased during treatment, with a tendency to reach a plateau earlier in higher GINA treatment steps. BUD/FORM maintenance and reliever therapy gave a significantly larger increase in FEV_1 _for all GINA treatment steps compared with higher maintenance dose ICS + SABA (mean FEV_1 _80.0-88.5 vs. 76.8-86.4) and for Steps 3 and 4 when compared with the same maintenance ICS/LABA + SABA group (mean FEV_1 _85.1-88.4 vs. 82.9-85.9). Mean FEV_1 _was similar in the BUD/FORM maintenance and reliever therapy group and the higher maintenance dose ICS/LABA + SABA group at the end of the study (84.8-89.1 vs. 84.7-89.1) (Figure 2).

### Reliever use

Patients in the higher GINA treatment steps at entry used more reliever medication (SABA or ICS/LABA as reliever) on average during the study than those in lower GINA treatment steps. Mean reliever use was significantly lower in patients receiving BUD/FORM maintenance and reliever therapy compared with higher maintenance dose ICS + SABA (0.737-1.165 vs. 1.100-1.734; P < 0.001) and same maintenance dose ICS/LABA + SABA (0.924-1.195 vs. 1.119-1.502; P < 0.001) at all GINA treatment steps apart from the Step 2 subgroup comparison with same maintenance dose ICS/LABA + SABA (0.834 vs. 0.928). When BUD/FORM maintenance and reliever therapy was compared with higher maintenance dose ICS/LABA + SABA there were no differences between study treatments in reliever use in either of the GINA treatment steps (0.874-1.105 vs. 0.889-1.143) (Additional file 1, Figure S1).

### Exacerbations

**Figure 3:**
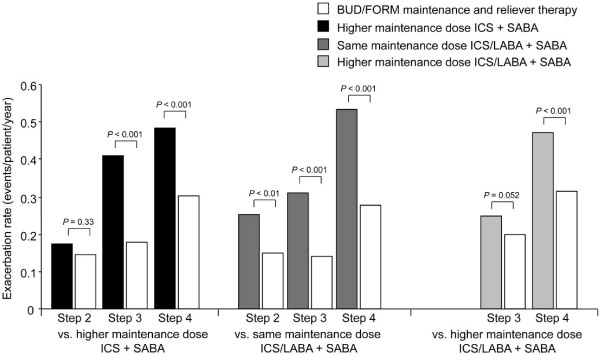
**Exacerbation rate by study treatment and GINA treatment step at study entry**. BUD/FORM = budesonide/formoterol; ICS = inhaled corticosteroid; LABA = long-acting β_2_-agonist; SABA = short-acting β_2_-agonist BUD/FORM maintenance and reliever therapy vs. higher maintenance dose ICS + SABA, exacerbation rate ratio (95% confidence interval): Step 2, 0.829 (0.570, 1.207); Step 3, 0.431 (0.353, 0.526); Step 4, 0.624 (0.512, 0.761). BUD/FORM maintenance and reliever therapy vs. same maintenance dose ICS/LABA + SABA, exacerbation rate ratio (95% confidence interval): Step 2, 0.583 (0.389, 0.874); Step 3, 0.455 (0.371, 0.558); Step 4, 0.519 (0.434, 0.620). BUD/FORM maintenance and reliever therapy vs. higher maintenance dose ICS/LABA + SABA, exacerbation rate ratio (95% confidence interval): Step 3, 0.795 (0.631, 1.002); Step 4, 0.665 (0.549, 0.807).

The rate of exacerbations during study treatment generally increased with increasing GINA treatment step at entry (Figure 3). BUD/FORM maintenance and reliever therapy significantly reduced the rate of exacerbations in patients in GINA treatment Steps 3 and 4 at entry compared with higher maintenance dose ICS + SABA (0.177 and 0.303 vs. 0.411 and 0.486) in all GINA treatment steps compared with same maintenance dose ICS/LABA + SABA (0.141-0.276 vs. 0.254-0.532) and in GINA treatment Step 4 patients compared with higher maintenance dose ICS/LABA + SABA (0.313 vs. 0.470) (Figure 3). In the remaining two groups, although exacerbations were numerically lower with BUD/FORM maintenance and reliever therapy the differences did not reach statistical significance (GINA treatment Step 2 patients vs. higher maintenance dose ICS + SABA [0.144 vs. 0.174], and GINA treatment Step 3 patients vs. higher maintenance dose ICS/LABA + SABA [0.198 vs. 0.250]) (Figure 3).

In all three comparisons, fewer patients receiving BUD/FORM maintenance and reliever therapy experienced a severe exacerbation compared with the fixed-dose controller regimens plus SABA: BUD/FORM maintenance and reliever therapy vs. higher maintenance dose ICS + SABA, 241 patients (12.9%) vs. 391 patients (20.9%), respectively [3,4]; BUD/FORM maintenance and reliever therapy vs. same maintenance dose ICS/LABA + SABA, 247 patients (12.2%) vs. 437 patients (21.4%), respectively [3,7]; and BUD/FORM maintenance and reliever therapy vs. higher maintenance dose ICS/LABA + SABA, 202 patients (9.0%) vs. 394 patients (11.7%), respectively [5,6].

**Figure 4:**
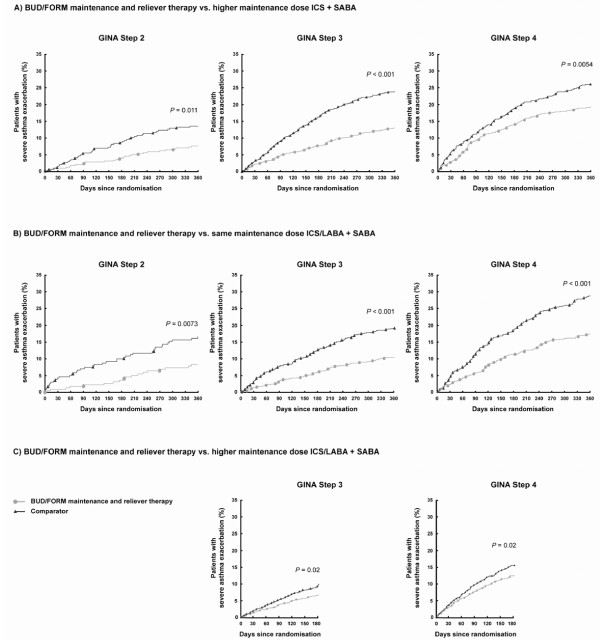
Time to first severe exacerbation by GINA treatment step at study entry. BUD/FORM = budesonide/formoterol; ICS = inhaled corticosteroid; LABA = long-acting β_2_-agonist; SABA = short-acting β_2_-agonist P-values refer to Cox model. BUD/FORM maintenance and reliever therapy vs. higher maintenance dose ICS + SABA, hazard ratio (95% confidence interval): Step 2, 0.545 (0.341, 0.870); Step 3, 0.507 (0.401, 0.642); Step 4, 0.701 (0.546, 0.900). BUD/FORM maintenance and reliever therapy vs. same maintenance dose ICS/LABA + SABA, hazard ratio (95% confidence interval): Step 2, 0.467 (0.268, 0.814); Step 3, 0.516 (0.406, 0.656); Step 4, 0.561 (0.449, 0.700). BUD/FORM maintenance and reliever therapy vs. higher maintenance dose ICS/LABA + SABA, hazard ratio (95% confidence interval): Step 3, 0.726 (0.554, 0.950); Step 4, 0.773 (0.620, 0.965).

In general, higher GINA treatment steps were associated with a shorter time to first event for all treatment groups (Figure 4). Within each GINA treatment step, the BUD/FORM maintenance and reliever therapy group showed a significantly prolonged time to first event compared with all three comparator fixed-dose maintenance regimens + SABA.

Relatively few events (<5% of patients) required hospital treatment or an ER visit during the study period in all groups, except among patients classified as GINA treatment Step 4 receiving same or higher maintenance dose ICS/LABA + SABA, for which 7.8% and 6.0% of patients required hospitalisation or an ER visit, respectively. However, most comparisons (rate and time to first exacerbation) favoured the BUD/FORM maintenance and reliever therapy group (Additional file 1, Table S4).

## Discussion

For ease of use, pharmacotherapy in asthma guidelines is arranged in treatment steps. Increasing treatment in patients who require additional treatment medication involves selecting an option from among the treatments on the same or a higher step. Treatments are stratified on the basis of randomised trials comparing their efficacy in patients qualifying for that treatment step with due regard to their therapeutic index, that is, weighing up benefit and safety. The results of this *post hoc *analysis of five large clinical studies provides clinically useful information on the relative efficacy and benefits of BUD/FORM maintenance and reliever therapy compared with high-dose ICS or fixed-dose ICS/LABA combinations plus SABA when introduced in patients previously prescribed treatments at Steps 2, 3 or 4 in the GINA report.

Satisfactory control, defined as Controlled or Partly Controlled asthma in the GINA report, was achieved in at least as many, or, for some comparisons and treatment steps, in a higher proportion of patients treated with BUD/FORM maintenance and reliever therapy compared with other fixed-dose maintenance regimens + SABA. For most comparisons, this was achieved with a lower mean dose of ICS and was associated with a lower risk of exacerbations (future risk). From the primary analyses of each of these trials, it is clear that lower exacerbation rates also resulted in significantly lower requirements for short courses of oral corticosteroids [3-7].

The superiority of BUD/FORM maintenance and reliever therapy over higher maintenance dose ICS alone with SABA as reliever in achieving satisfactory control (defined as Controlled or Partly Controlled by GINA) for patients previously prescribed Step 2 treatment is an important observation. This analysis suggests that using BUD/FORM both as maintenance and reliever for patients not controlled at this treatment step offers significant benefit. Secondly, BUD/FORM maintenance and reliever therapy at Steps 3 and 4 is also superior to fixed-dose ICS/LABA given at the same maintenance dose of ICS in achieving reductions in exacerbations, although it is only in patients at Step 4 that a statistically significant advantage is seen in current clinical control. Finally, BUD/FORM maintenance and reliever therapy achieves only similar levels of current clinical control to a higher maintenance dose of ICS/LABA but retains its advantage in reducing exacerbations (only statistically significant in patients on Step 4), making it an attractive option in patients on this step for reducing exposures to inhaled and systemic corticosteroids.

These results are supported by the changes in FEV_1 _and as-needed use of reliever medication. Improvements in FEV_1 _were significantly greater with BUD/FORM maintenance and reliever therapy for all comparisons other than at Step 2 when compared with same maintenance dose ICS/LABA + SABA and with a higher maintenance dose of ICS/LABA + SABA. The reduction in reliever use was significant in the comparison with a higher maintenance dose ICS + SABA in Steps 2-4 and Steps 3 and 4 when compared with same maintenance dose ICS/LABA + SABA.

A further finding of the analysis is the relationship between treatment step and other indicators of asthma severity. A recent American Thoracic Society/European Respiratory Society Task Force Report on asthma severity and control states that the level of treatment required to achieve and/or maintain control is the most consistent indicator of severity [11]. In our analysis, patients on higher treatment steps at entry were older (less than 10 years' mean difference between Steps 2 and 4), possibly indicating increasing treatment requirements associated with lifelong asthma, and there was a trend towards lower FEV_1 _and higher symptom scores during run-in. Consistent with this, the proportion of patients with GINA-defined Controlled asthma and Controlled or Partly Controlled asthma at study end decreased with increasing GINA treatment step at entry, irrespective of study treatment. These results are also consistent with those of the Gaining Optimal Asthma Control (GOAL) study in which the proportion of patients achieving target levels of control decreased with increasing severity of asthma categorised according to prior treatment. This was in spite of escalation of ICS or fixed-dose ICS/LABA to maximum recommended levels for a prolonged period [12].

The limitations of this study include that it is a *post hoc *analysis; patients were not randomised according to GINA treatment step in each study. The assessment of GINA-defined asthma control was also *post hoc *and based upon data derived from patient diary cards; however, the method that was employed and its usefulness have been discussed in a previous report [9]. A further limitation is that, although in all the studies asthma symptoms and reliever use during run-in confirmed that patients were uncontrolled at entry, in three of the five studies some medications had been withdrawn during this phase, resulting in worse control than at recruitment. While this will have served to emphasise the benefits of subsequent treatments, it would not have favoured any of the treatments. In only one study was the control state known prior to run-in, and in this study the five-item Asthma Control Questionnaire score was higher than 2.1, confirming that the majority of those recruited were uncontrolled [7]. Furthermore, the numbers of patients on Step 2 treatment and the relatively low exacerbation rates in this subgroup weakens the confidence of the estimate. Finally, because of the relatively short duration of the studies (6-12 months), we have not attempted to study other aspects of future risk such as lung function decline or the adverse effects of treatment. However, a recent combined analysis of these five and one additional study comparing BUD/FORM maintenance and reliever therapy with fixed-dose alternatives confirmed that the former was associated with fewer asthma-related serious adverse events and discontinuations during the studies and with no increased risk of deaths or cardiac-related serious adverse events [13].

## Conclusions

In summary, this analysis confirms that the BUD/FORM maintenance and reliever therapy approach is a highly effective option for patients requiring treatment adjustments across Steps 2 to 4 in the GINA treatment guidelines. The as-needed use of BUD/FORM for relief is obviously of greatest benefit to patients in each treatment step whose asthma control is not optimal, either because they are undertreated or because their asthma is more refractory (severe). The reduction in such patients' rates of asthma exacerbations, with attendant reduction in need for systemic corticosteroids, is a useful advantage and, although not examined in this analysis, might be associated with reductions in risk of adverse effects of treatment, particularly in the long term.

## Conflicts of interest

EDB has received honoraria for consulting, speaking at scientific meetings and participating in advisory boards for AstraZeneca. His institution has received grants for participation in clinical trials. TH has received honoraria from AstraZeneca for advisory work and presentations. SQ has been on advisory boards for and has received speaker's honoraria from AstraZeneca, GlaxoSmithKline, MSD, Novartis, Almirall, Altana, Chiesi and Pfizer. HR has been on advisory boards for AstraZeneca, GlaxoSmithKline and Novartis, has received speaker's honoraria from GlaxoSmithKline, AstraZeneca, Merck and Getz Pharma, has provided consultancy services for Biota and GlaxoSmithKline, and has received research funding from GlaxoSmithKline and AstraZeneca. RB has received reimbursement for attending scientific conferences and/or fees for speaking and/or consulting from AstraZeneca, Boehringer Ingelheim, Chiesi, GlaxoSmithKline, Novartis, Nycomed and Pfizer. The Pulmonary Department at Mainz University Hospital received financial compensation for services performed during participation in clinical trials organised by various pharmaceutical companies. MH has relationships with drug companies including AstraZeneca, Chiesi, GlaxoSmithKline, MSD, Novartis, Nycomed and Pfizer. In addition to being investigator in trials involving these companies, relationships include consultancy services and membership of scientific advisory boards. CJ is employed by the Woolcock Institute of Medical Research. The Institute receives funding for its ongoing education and research programs from AstraZeneca and GlaxoSmithKline and conducts clinical trials for these and other pharmaceutical companies under contract. She receives no monies directly through these sources or funding through the Co-operative Research Centre for Asthma, a collaborative research programme funded jointly by the Australian Government and Industry partners. In the last 3 years CJ has received reimbursement for Advisory Board Membership, consultancy and speakers fees in from Altana, Astra Zeneca, GlaxoSmithKline, Hunter Immunology, Novartis, Nycomed and Tyrian Diagnostics. PO'B has been on advisory boards for AstraZeneca, GlaxoSmithKline, Merck, Nycomed, Topigen and Wyeth and has received lecture fees from these and other pharmaceutical companies including Chiesi and Ono Pharma. In addition, he has received grants for research studies from AstraZeneca, Genentech, GlaxoSmithKline, MedImmune, Merck, Pfizer, Topigen and Wyeth. MRS holds an Endowed chair in Respiratory Epidemiology jointly endowed by AstraZeneca and McMaster University. He has received research funding from pharmaceutical companies including Merck Sharp Dome and AstraZeneca. He has acted as a consultant or advisory board member to Merck Sharp Dome, Novartis, and AstraZeneca. OÖ, SP, GSE are employed by AstraZeneca and own shares in AstraZeneca.

## Authors' contributions

All authors read and approved the final manuscript. EDB was an investigator in three of the clinical trials, and was involved in the study design, analysis and interpretation of data and the lead in drafting the manuscript. TH contributed to the design, interpretation of results and drafting the manuscript. SQ has participated in planning and discussion of the manuscript. HR participated in the study design, analysis and interpretation of data and the writing of the manuscript. RB contributed to the planning of the analyses, commented on the data/results of the analyses and provided input to the interpretation of the data and also contributed to the final manuscript. MH contributed to data analysis and approved the manuscript. CJ participated in the study design, analysis and interpretation of data and the writing of the manuscript. SP and OÖ contributed to the statistical analysis plan, performed the statistical analyses and contributed to the writing of the manuscript. PO'B contributed to the development of the research strategy, evaluation and analysis of the data and to the writing of the manuscript. MRS participated in data evaluation, editing of draft manuscripts, and approval of the final manuscript. GSE was involved in the study design, analysis and interpretation of data and the drafting of the manuscript.

## Supplementary Material

Additional file 1 **Overall asthma control achieved with budesonide/formoterol maintenance and reliever therapy for patients on different treatment steps - additional data **Additional information providing supplementary trial details, hospitalisation and ER rates and reliever use data Click here for file
