# Supplementary material for: Overall asthma control achieved with budesonide/formoterol maintenance and reliever therapy for patients on different treatment steps
Source: Respir Res. 2011 Apr 4;12(1):38. doi: 10.1186/1465-9921-12-38 (PMC3082240; doi:10.1186/1465-9921-12-38)

**Overall asthma control achieved with budesonide/formoterol maintenance and reliever therapy for patients on different treatment steps – additional data**

# Methods

## Studies and population

This retrospective analysis included all long-term, double-blind, randomised, parallel-group clinical studies (6–12 months in duration) investigating the efficacy of budesonide/formoterol (BUD/FORM) maintenance and reliever therapy (Symbicort SMART®, AstraZeneca, Lund, Sweden) versus the following comparator therapies: higher maintenance dose BUD plus short-acting β_2_-agonist (SABA) as needed, same maintenance dose BUD/FORM (Symbicort®, AstraZeneca, Lund, Sweden) plus SABA as needed and higher maintenance dose BUD/FORM or salmeterol/fluticasone (Seretide™, GlaxoSmithKline, Uxbridge, UK) plus SABA as needed, with exacerbation as a primary endpoint. Study interventions, patient numbers and the mean inhaled corticosteroid (ICS) dose for each study are shown in Additional file 1, Table S1.

The studies by O’Byrne *et al*. [3] and Scicchitano *et al*. [4] compared BUD/FORM maintenance and reliever therapy (80/4.5 μg twice daily [bid] [3] and 160/4.5 μg two inhalations once daily [4]) with higher maintenance dose BUD (Pulmicort®, AstraZeneca, Sweden; 320 μg bid [3], 160 μg two inhalations bid [4]) plus terbutaline as needed (Bricanyl®, AstraZeneca, Sweden; 0.4 mg/inhalation).

In the study by Rabe *et al*. [7] and in a further treatment arm in the O’Byrne *et al*. study [3], the comparator arm was the same maintenance dose of ICS/long-acting β_2_-agonist (LABA) as used in the BUD/FORM maintenance and reliever therapy arm (160/4.5 μg bid [7] and 80/4.5 μg bid, respectively) plus terbutaline (0.4 mg) [3,7] as needed. In the study by Rabe *et al*. [7] there was an additional treatment arm that included the same maintenance dose of ICS/LABA as used in the BUD/FORM maintenance and reliever therapy arm (160/4.5 μg bid) plus formoterol 4.5 μg (Oxis®, AstraZeneca, Sweden) as needed. However, only data from the terbutaline as-needed arm were included in the present analysis; data from the formoterol as-needed arm were excluded.

Data from Kuna *et al*. [5] and Bousquet *et al*. [6] comparing budesonide/formoterol maintenance and reliever therapy (160/4.5 μg bid [5] and 160/4.5 μg two inhalations bid [6]) with a higher maintenance dose ICS in combination with LABA [5] (BUD/FORM 320/9 μg bid or salmeterol/fluticasone 25/125 μg two inhalations bid [5]; salmeterol/fluticasone 50/500 μg bid [6]) plus terbutaline (0.4 mg/inhalation) were also pooled.

## Stratification by GINA treatment steps at entry

Data collected at study entry were used to classify each patient’s pre-study asthma medication as fitting Global Initiative for Asthma (GINA) Step 2, 3 or 4, based on ICS dose and use of other maintenance medication. ICS doses were classified as high, medium or low according to the breakpoints for adults and children shown in Additional file 1, Table S2. Additional file 1, Table S3 shows the proportion of patients classified in each GINA step and the pre-entry medication for each study. In all comparisons of BUD/FORM maintenance and reliever therapy with higher maintenance dose ICS/LABA + SABA, seven Step 2 patients from Kuna *et al.* and Bousquet *et al.* [5,6] were pooled with the Step 3 patients because the number of patients was not sufficient for analysis as a separate Step 2 group. However, a stability analysis showed that their inclusion in Step 3 did not notably affect the results.

## Asthma control as defined by GINA criteria

For each of the five studies, GINA-defined asthma control was determined for each week of the study (here we report values at run-in and at the last week of study treatment) by evaluation of the exacerbation data and patient diary card data. The following subcriteria were considered: i) night-time symptoms or awakenings per week (none allowed); ii) daytime symptoms (at most 2 days/week); iii) reliever use (at most 2 days/week with no more than two inhalations each day, or alternatively up to four inhalations on no more than 1 day during the week); iv) morning peak expiratory flow (PEF) (≥ 80% predicted normal before medication on every morning of the week); and v) activity limitation as determined from daytime symptoms (daytime symptom score < 2 on every day of the week, where: 0 = no asthma symptoms; 1 = aware of asthma symptoms but can easily tolerate them; 2 = asthma causes enough discomfort to cause problems with normal activities; 3 = unable to do normal activities due to symptoms).

A week for any patient was considered Controlled if, during that week, all five of the diary card subcriteria were controlled and no severe exacerbation was recorded. The week was defined as Partly Controlled if any one or two of the subcriteria were uncontrolled and no exacerbation was recorded. If the week had three or more uncontrolled subcriteria and/or an exacerbation, the week was termed Uncontrolled. Control of subcriteria had to be positively demonstrated by diary data; if data were missing, subcriteria were considered Uncontrolled.

## Exacerbations

There were minor differences in the definition of exacerbations between the studies. O’Byrne *et al.* [3] defined severe asthma exacerbations as a deterioration in asthma resulting in hospitalisation/emergency room (ER) treatment, oral steroid treatment (or an increase in ICS [via a separate inhaler] and/or other additional treatment for children aged 4–11 years) or morning PEF of ≤ 70% of baseline on two consecutive days. Scicchitano *et al.* [4] defined a severe exacerbation as asthma worsening resulting in hospitalisation or ER treatment, the need for systemic steroids or a fall in morning PEF to ≤ 70% of baseline on two consecutive days. Rabe *et al*. [7], Kuna *et al.* [5] and Bousquet *et al.* [6] used the following definition for a severe exacerbation: a deterioration in asthma resulting in emergency treatment or hospitalisation or the need for oral steroids for 3 days or more (as judged by the investigator).

Therefore, for this pooled analysis, a standardised definition of a worsening of asthma symptoms requiring an oral steroid course of at least 3 days’ duration, an ER visit or a hospitalisation was used for an exacerbation, incorporating the common elements from all studies.

# Results

## Exacerbations

The hospitalisation rates were significantly lower with BUD/FORM maintenance and reliever therapy for GINA Step 3 patients compared with higher maintenance dose ICS + SABA and for GINA Step 4 patients compared with same and higher maintenance dose ICS/LABA + SABA (Additional file 1, Table S4). There was a numerical disadvantage of BUD/FORM in Step 2 patients compared with higher maintenance dose ICS + SABA and same maintenance dose ICS/LABA + SABA, based on relatively few events (9 vs. 4 and 7 vs. 4 events, respectively). Both these comparisons are affected by two patients receiving BUD/FORM maintenance and reliever therapy having three and four hospitalisations or ER visits each.

The results for time to first hospitalisation or ER visit show a similar pattern to that observed for time to first severe exacerbation. There was a significant advantage of BUD/FORM maintenance and reliever therapy in GINA Step 3 patients compared with higher maintenance dose ICS + SABA and higher maintenance dose ICS/LABA + SABA and in GINA Step 4 patients compared with same maintenance dose ICS/LABA + SABA (Additional file 1, Table S4).

## Reliever use

Mean reliever use during study treatment was generally greater in patients in the higher GINA treatment steps vs. patients in lower GINA treatment steps. For therapy comparison, mean reliever use was lower in patients receiving BUD/FORM maintenance and reliever therapy compared with higher maintenance dose ICS + SABA (0.737–1.165 vs. 1.100–1.734; P < 0.001) at all GINA steps and compared with same maintenance dose ICS/LABA + SABA (0.924–1.195 vs. 1.119–1.502; P < 0.001) at Steps 3 and 4. Reliever use was comparable with BUD/FORM maintenance and reliever therapy vs. higher maintenance dose ICS/LABA + SABA (0.874–1.105 vs. 0.889–1.143) at Step 3 or 4 (Additional file 1, Figure S1).

When reliever use is grouped it can be seen that the majority of patients used less than 0.5 inhalations/day and only about 10–20 % used more than 2 inhalations per day. As might be expected more patients on Step 4 at entry used more than two puffs/day; in those using BUD/FORM as maintenance and reliever therapy, reliever use tended to be lower than in those on comparator treatments (with SABA as reliever) (Additional file 1, Figure S2).

#### Table S1: Summary of the budesonide/formoterol maintenance and reliever therapy clinical trials used in this retrospective analysis

|  | **Study duration, months** | **Study intervention** | **No. patients** | **Mean ICS µg/day (BDP equiv.)** *****^†^ |
| --- | --- | --- | --- | --- |
| **Comparison with higher maintenance dose ICS maintenance therapy plus SABA** | | | | |
| O’Byrne *et al.* [3] SD-039-0673 | 12 | BUD/FORM maintenance + reliever (80/4.5 µg bid)  BUD (320 µg bid) plus SABA | 925 926 | 240 (375) 640 (1000) |
| Scicchitano *et al.* [4] SD-039-0668 | 12 | BUD/FORM maintenance + reliever (2 x 160/4.5 µg qd) BUD (2 x 160 µg bid) plus SABA | 947 943 | 466 (728) 640 (1000) |
| **Comparison with same maintenance dose ICS/LABA maintenance therapy plus SABA** | | | | |
| O’Byrne *et al.* [3]  SD-039-0673 | 12 | BUD/FORM maintenance + reliever (80/4.5 µg bid)  BUD/FORM (80/4.5 µg bid) plus SABA | 925 909 | 240 (375) 160 (250) |
| Rabe *et al.* [7] SD-039-0734 | 12 | BUD/FORM maintenance + reliever (160/4.5 µg bid)  BUD/FORM (160/4.5 µg bid) plus reliever^‡^ | 1113 2281 | 483 (755) 320 (500) |
| **Comparison with higher maintenance dose ICS/LABA maintenance therapy plus SABA** | | | | |
| Kuna *et al.* [5] SD-039-0735 | 6 | BUD/FORM maintenance + reliever (160/4.5 µg bid)  BUD/FORM (320/9 µg bid) plus SABA  SAL/FLU (2 x 25/125 µg bid) plus SABA | 1107 1105 1123 | 483 (755) 640 (1000) 500 (1000) |
| Bousquet *et al.* [6] NCT00242775 | 6 | BUD/FORM maintenance + reliever (2x 160/4.5 µg bid) SAL/FLU (50/500 µg bid) plus SABA | 1154 1155 | 792 (1238) 1000 (2000) |

BDP = beclomethasone dipropionate; bid = twice daily; BUD = budesonide; FLU = fluticasone; FORM = formoterol; ICS = inhaled corticosteroid; LABA = long-acting β_2_-agonist; qd = once daily; SABA = short-acting β_2_-agonist; SAL = salmeterol.

* Microgram dose of ICS is stated as prescribed regular daily dose. In patients on BUD/FORM maintenance and reliever therapy, the additional mean dose of ICS taken as needed as recorded in the patient diary has been added to the regular daily dose.

^†^ Mean ICS doses converted to BDP equivalents based on GINA guidelines [2].

^‡^ Only data from the terbutaline as-needed arm were included in the present analysis (N = 1141); data from the formoterol as-needed arm (N = 1140) were excluded.

#### Table S2. Inhaled corticosteroid dose classification thresholds (upper limits) [2]

|  | **Dose classification thresholds (μg)** | | | |
| --- | --- | --- | --- | --- |
| **Inhaled corticosteroid** | **Low** | | **Medium** | |
|  | Adult | Child | Adult | Child |
| Beclomethasone dipropionate | ≤ 500 | ≤ 200 | ≤ 1000 | ≤ 400 |
| Budesonide | ≤ 400 | ≤ 200 | ≤ 800 | ≤ 400 |
| Flunisolide | ≤ 1000 | ≤ 750 | ≤ 2000 | ≤ 1250 |
| Fluticasone | ≤ 250 | ≤ 200 | ≤ 500 | ≤ 500 |
| Triamcinolone | ≤ 1000 | ≤ 800 | ≤ 2000 | ≤ 1200 |

Child = < 12 years of age.

#### Table S3. Summary of pre-entry medication by study

|  |  | Bousquet *et al.* N = 2304 | Kuna  *et al.* N = 3321 | O’Byrne  *et al.* N = 2753 | Rabe  *et al.* N = 2245 | Scicchitano *et al.* N = 1889 | Total  N = 12512 |
| --- | --- | --- | --- | --- | --- | --- | --- |
| GINA treatment step, n (%) | Step 2 | 4 (< 0.5) | 3 (< 0.5) | 698 (25) | 18 (1) | 314 (17) | 1037 (8) |
|  | Step 3 | 1245 (54) | 1737 (52) | 1383 (50) | 1158 (52) | 829 (44) | 6352 (51) |
|  | Step 4 | 1055 (46) | 1581 (48) | 672 (24) | 1069 (48) | 746 (39) | 5123 (41) |
| Inhaled corticosteroid, n (%) | Low | 257 (11) | 6 (<0.5) | 902 (33) | 343 (15) | 456 (24) | 1964 (16) |
|  | Medium | 1720 (75) | 2781 (84) | 1663 (60) | 1519 (68) | 1008 (53) | 8691 (69) |
|  | High | 327 (14) | 534 (16) | 188 (7) | 383 (17) | 425 (22) | 1857 (15) |
| Long-acting β_2_-agonist,  n (%) | | 1262 (55) | 1525 (46) | 759 (28) | 1319 (59) | 820 (43) | 5685 (45) |
| Xanthines, n (%) | | 70 (3) | 201 (6) | 134 (5) | 193 (9) | 81 (4) | 679 (5) |
| Leukotriene receptor antagonist, n (%) | | 54 (2) | 101 (3) | 86 (3) | 71 (3) | 54 (3) | 366 (3) |
| Systemic selective β_2_-agonist, n (%) | | 39 (2) | 16 (<0.5) | 34 (1) | 43 (2) | 10 (1) | 142 (1) |
| Anticholinergic, n (%) | | 15 (1) | 68 (2) | 24(1) | 39 (2) | 48 (3) | 194 (2) |
| Cromone, n (%) | | 1 (<0.5) | 0 | 2 (<0.5) | 0 | 0 | 3 (<0.5) |

#### Table S4.

#### A) Comparison of rate of hospitalisations or ER visits by GINA treatment step at entry and treatment by Poisson regression model

| **BUD/FORM maintenance and reliever therapy vs.** | **Treatment ratio according to GINA treatment step at entry** | | |
| --- | --- | --- | --- |
|  | **Step 2** | **Step 3** | **Step 4** |
| Higher maintenance dose ICS + SABA | 2.114  (0.651, 6.864)  P = 0.21 | 0.433  (0.220, 0.851)  P = 0.015 | 0.841  (0.455, 1.553)  P = 0.58 |
| Same maintenance dose ICS/LABA + SABA | 1.948  (0.570, 6.655)  P = 0.29 | 0.723  (0.492, 1.063)  P = 0.099 | 0.529  (0.366, 0.766)  P < 0.001 |
| Higher maintenance dose ICS/LABA + SABA | N/A | 0.720  (0.514, 1.009)  P = 0.056 | 0.708  (0.527, 0.951)  P = 0.022 |

#### B) Comparison of time to first hospitalisation or ER visit by GINA treatment step at entry and treatment by Cox proportional hazard model

| **BUD/FORM maintenance and reliever therapy vs.** | **Treatment ratio according to GINA treatment step at entry** | | |
| --- | --- | --- | --- |
|  | **Step 2** | **Step 3** | **Step 4** |
| Higher maintenance dose ICS + SABA | 1.260  (0.282, 5.629)  P = 0.76 | 0.320  (0.137, 0.748)  P = 0.0086 | 0.980  (0.490, 1.961)  P = 0.96 |
| Same maintenance dose ICS/LABA + SABA | 0.759  (0.127, 4.543)  P = 0.76 | 0.662  (0.428, 1.023)  P = 0.063 | 0.548  (0.358, 0.837)  P = 0.0055 |
| Higher maintenance dose ICS/LABA + SABA | N/A | 0.627  (0.421, 0.931)  P = 0.021 | 0.835  (0.594, 1.175)  P = 0.3 |

BUD/FORM = budesonide/formoterol; ICS = inhaled corticosteroid; LABA = long-acting β_2_-agonist; SABA = short-acting β_2_-agonist.

Data presented are treatment ratio (95% confidence interval) and P-value.

#### Figure S1. Mean reliever use by GINA step at study entry


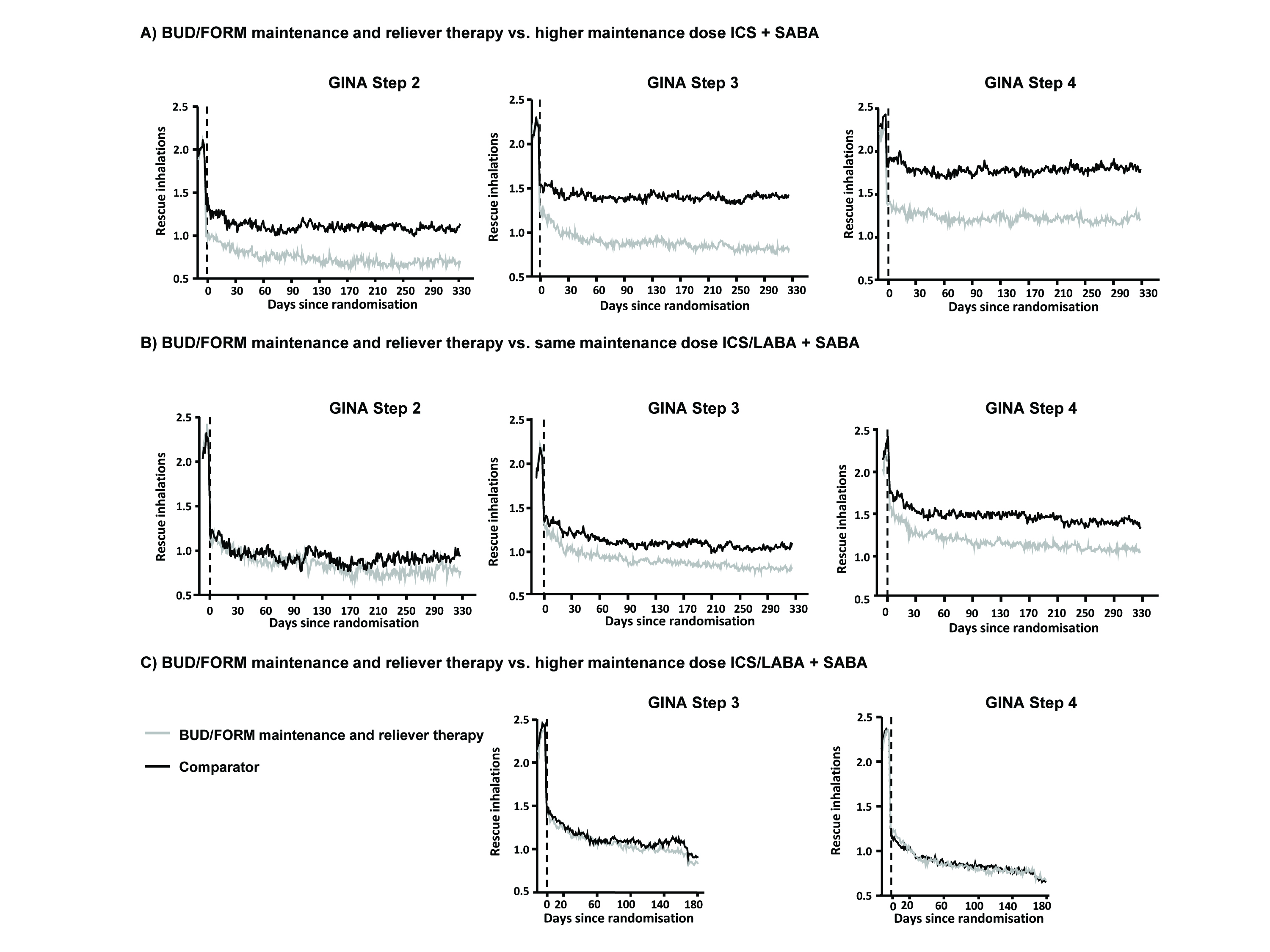


**Figure S2. Grouped mean reliever use by GINA step at study entry**


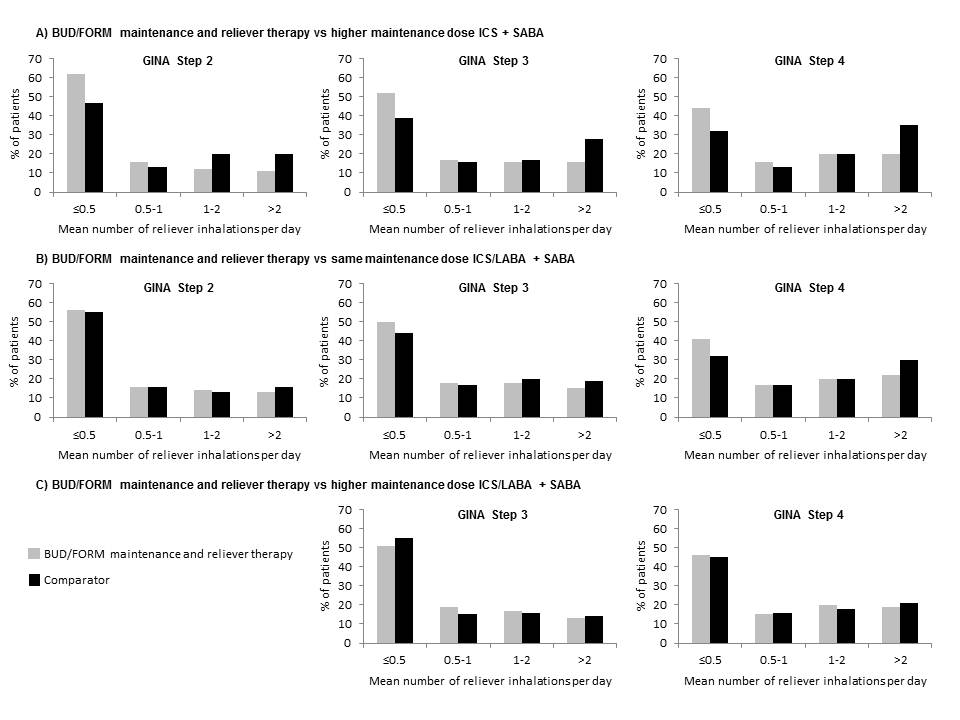

Supplement: Additional file 1 — Overall asthma control achieved with budesonide/formoterol maintenance and reliever therapy for patients on different treatment steps - additional data Additional information providing supplementary trial details, hospitalisation and ER rates and reliever use data [file 1465-9921-12-38-S1.DOC]
